# Supplementary material for: Saccharomyces boulardii (CNCM I-745) improves intestinal damage in sepsis by remodeling intestinal flora structure
Source: Front Cell Infect Microbiol. 2025 Jul 31;15:1602792. doi: 10.3389/fcimb.2025.1602792 (PMC12350329; doi:10.3389/fcimb.2025.1602792)
Supplement: Supplementary file 1 [file DataSheet1.zip › summary/summary/7_Differential_analysis/1_diff_abundance/6_Genus/Group.DW4_vs_DW0/Group.DW4_vs_DW0_diff_barplot.pdf]

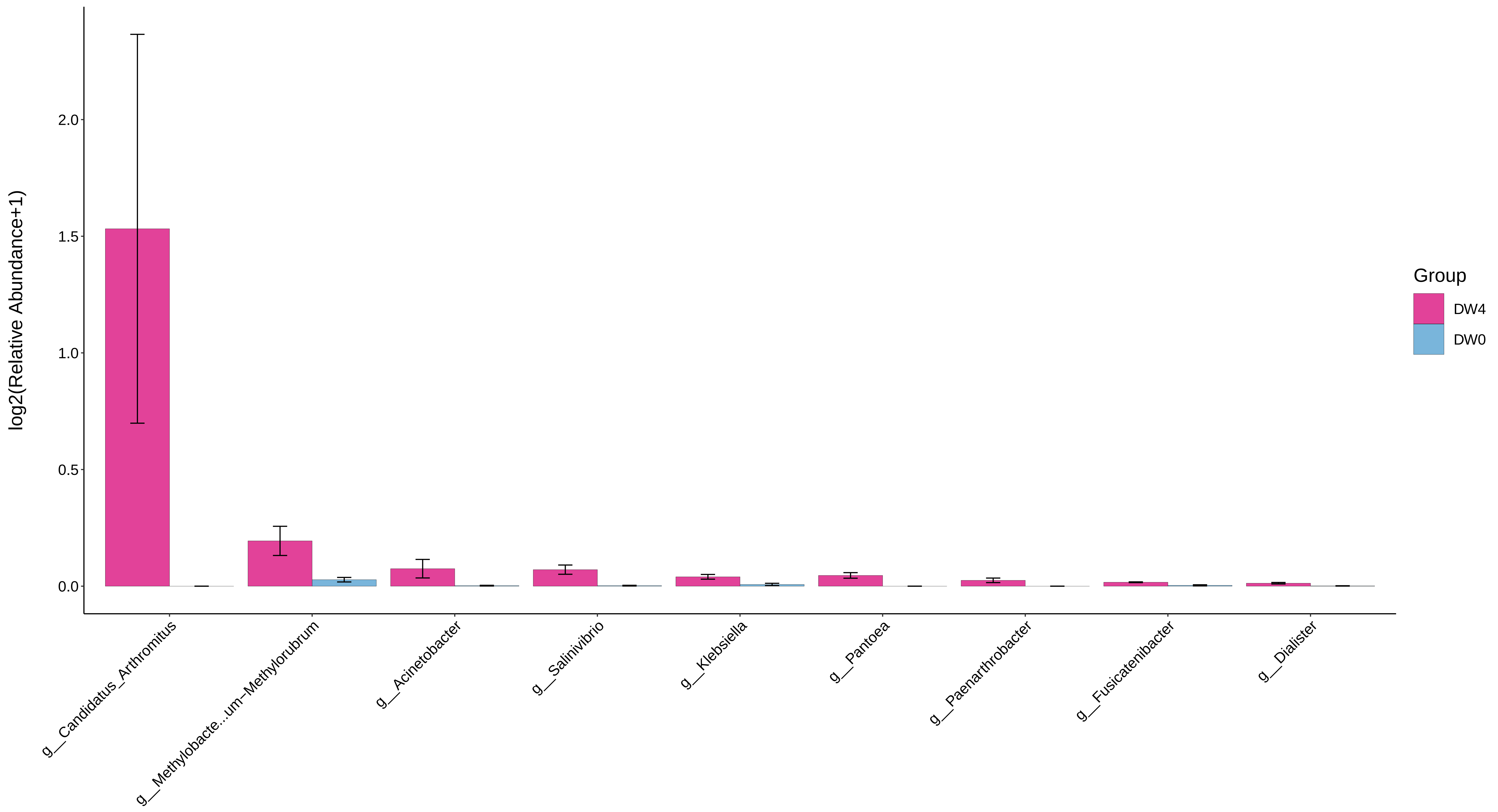

log2(Relative Abundance+1)
0.0
0.5
1.0
1.5
2.0
Group
DW4
DW0
g__Candidatus_Arthromitus
g__Methylobacte...um−Methylorubrum
g__Acinetobacter
g__Salinivibrio
g__Klebsiella
g__Pantoea
g__Paenarthrobacter
g__Fusicatenibacter
g__Dialister
